# An Innovative Model of Stroke Care for Rapid Assessment and Discharge of Patients With Transient Ischemic Attack and Stroke in Northeastern Ontario: Protocol for the Implementation and Evaluation of MOTIVE (Mobile Transient Ischemic Attack and Stroke With Adaptive Workflow) Team

**DOI:** 10.2196/93315

**Published:** 2026-07-03

**Authors:** Venkadesan Rajendran, Chantal Liddard, Lisa Zeman, Susan Bursey, Ravinder-Jeet Singh

**Affiliations:** 1 Health Sciences North Sudbury, ON Canada; 2 NOSM University Sudbury, ON Canada

**Keywords:** acute stroke, transient ischemic attack, interdisciplinary assessment, quality improvement, length of stay

## Abstract

**Background:**

Patients presenting to the emergency department (ED) with transient ischemic attack (TIA) or stroke, as well as admitted patients who develop stroke symptoms in acute nonstroke units, are commonly transferred to stroke units, where trained interdisciplinary teams provide comprehensive assessments and discharge planning. However, the lack of integrated interdisciplinary stroke assessments in the ED and acute nonstroke unit care settings has contributed to inefficient patient flow and capacity pressure, prolonged hospital length of stay (LOS), and delayed discharge. Prior models have emphasized rapid outpatient TIA or stroke prevention clinics and ED observation pathways; these approaches have largely focused on expedited medical diagnosis and treatment, with limited attention to coordinated interdisciplinary functional assessment and discharge planning to facilitate early discharge, especially from the ED and nonstroke units, reduce readmissions, and support rehabilitation.

**Objective:**

This protocol outlines the implementation and prospective evaluation of the MOTIVE (Mobile Transient Ischemic Attack and Stroke With Adaptive Workflow) team, an interdisciplinary mobile service operational within the ED and inpatient nonstroke units at Health Sciences North, a regional stroke center in Northeastern Ontario. The service aims to deliver prompt medical and functional assessments to facilitate early decision-making and discharge planning. The objectives are to (1) reduce avoidable ED admissions for patients with TIA and minor stroke, (2) decrease hospital LOS, (3) maintain patient safety as measured by 30-day readmission rates, and (4) enhance patient and caregiver experience.

**Methods:**

This single-center, prospective, uncontrolled, before-and-after quality improvement study will use control charts to evaluate temporal changes in outcomes. The MOTIVE project will follow the Institute for Healthcare Improvement Model for Improvement and will be reported in accordance with the SQUIRE (Standards for Quality Improvement Reporting Excellence) 2.0 guidelines. The preimplementation period (fiscal year 2022) will serve as the baseline period. The postimplementation evaluation will cover 12 months following the full implementation of the MOTIVE team, with iterative refinement guided by Plan-Do-Study-Act cycles.

**Results:**

This is a study protocol; therefore, the results are not yet available. Planned analyses will include run charts and control charts to evaluate temporal trends, alongside preimplementation and postimplementation comparisons. Outcomes will comprise primary measures (ED-to-inpatient admission rate, acute LOS, and avoided bed-day costs), process measures (time to interdisciplinary assessment, magnetic resonance imaging wait time, and proportion of patients triaged within 24 h), and balancing measures (30-day readmission, outpatient therapy services wait time, stroke prevention clinic referral volume and wait time, and patient and caregiver experience).

**Conclusions:**

This innovative MOTIVE model is anticipated to improve stroke care efficiency, patient outcomes, and patient and caregiver experience through timely interdisciplinary assessments and support earlier discharge for select patients. The findings of this study will inform the feasibility and scalability of this model in similar health care contexts.

**Trial Registration:**

OSF Registries osf.io/2jybu; https://osf.io/2jybu

**International Registered Report Identifier (IRRID):**

DERR1-10.2196/93315

## Introduction

Stroke is one of the leading causes of mortality and long-term disability in Canada, resulting in physical, cognitive, visual, and communication impairments. A stroke occurs approximately every 5 minutes, affecting more than 878,000 Canadians, a figure projected to rise with the aging population. The annual economic burden exceeds CAD $3.6 billion (CAD $1=US $0.71 as of June 23, 2026), including direct health care costs and productivity losses [1-4]. Up to 25% of strokes are preceded by transient ischemic attacks (TIAs), representing a critical opportunity for secondary prevention. Evidence demonstrates that timely specialist assessment of TIA or minor stroke reduces the risk of major stroke by 80% to 90%, thereby decreasing mortality and costs. Accordingly, the Canadian Stroke Best Practice Recommendations (CSBPR) recommend interdisciplinary evaluation of patients with suspected TIA or minor stroke prior to discharge [4].

For patients experiencing acute stroke, the CSBPR further recommend rehabilitation screening within 48 hours by an interdisciplinary team including a stroke physician, physiotherapist, occupational therapist, and speech-language pathologist to guide individualized rehabilitation, prevent secondary complications, and support appropriate triage to intensive inpatient rehabilitation for those with moderate to severe multidomain impairment or to community-based services for those with milder deficits [5].

Despite robust evidence and national standards, implementation remains inconsistent in both urban and rural settings. Health Sciences North (HSN), the regional stroke center for Northeastern Ontario, evaluates approximately 425 emergency department (ED) presentations annually for suspected TIA or stroke. Among all TIA and stroke ED presentations, the current admission rate is 94%, with a mean acute length of stay (LOS) of 11.2 days.

Although physician assessments are typically timely, comprehensive interdisciplinary evaluations, including the recommended 48-h rehabilitation screening, are often delayed until a patient is transferred to the 12-bed Acute Stroke Unit (ASU). Approximately 20% of the patients are admitted to nonstroke units from the ED or other (nonstroke) units. For patients who develop stroke symptoms on nonstroke units, interdisciplinary evaluation may be delayed by more than 7 days from admission; this pattern is observed in approximately 20% of admitted patients and is attributed to the absence of a stroke-specific interdisciplinary team in those units.

To understand these gaps, the quality improvement (QI) team conducted a root cause analysis using a cause-and-effect diagram (Multimedia Appendix 1) and a best practice gap analysis (Table 1). Three interrelated system drivers were identified: (1) a provincial quality-based procedure funding model that ties reimbursement and specialized staffing to maintaining ≥75% occupancy of geographically fixed ASU beds, creating a strong financial incentive for inpatient admission relative to ED or non-ASU–based care [6]; (2) the resulting inability to deploy interdisciplinary stroke resources outside the ASU; and (3) the absence of a magnetic resonance imaging (MRI) prioritization framework, leading to diagnostic delays of up to 5 days and reinforcing precautionary admissions.

**Table 1:** Best practice gap analysis in acute stroke and transient ischemic attack (TIA) at Health Sciences North (HSN).

Domain	Canadian Stroke Best Practice Recommendation	Current performance at HSN	Identified gap
ED^a^ triage and assessment	Rapid triage and assessment in the ED by a specialized interdisciplinary stroke team	No specialized interdisciplinary stroke team servicing the ED	Lack of early involvement of interdisciplinary stroke expertise; delay in accessing stroke care
Hospital admission practices	Approximately 70%-80% of patients with stroke or TIA require hospital admission	94% admission rate from the HSN ED	Overadmission of TIA and minor stroke cases (up to 24%), contributing to bed occupancy pressures and reduced patient flow on the ASUb
Access to diagnostic imaging	Patients with TIA and minor stroke should have rapid access to diagnostic imaging (CTc or CTAd, with MRIe when indicated)	MRI wait times up to 5 d TIA or minor stroke patients are frequently admitted primarily to access imaging No prioritization framework for stroke imaging beyond Code Stroke cases	Delayed diagnostic imaging (up to 5 d), resulting in potentially avoidable admissions
Interdisciplinary stroke care	Interdisciplinary stroke team assessment within 48 h of admission	Approximately 20% of admitted stroke patients are managed on non-ASU units Specialized interdisciplinary stroke assessment begins only after transfer to ASU	Delayed access (up to 7 d) to stroke-specific interdisciplinary assessment and therapy initiation for patients on non-ASU units
Rehabilitation planning	Determination of the postacute rehabilitation pathway within 72 h of admission	Decisions are frequently delayed, particularly for patients admitted to non-ASU units	Delayed rehabilitation initiation (up to 7 d), compounded by limited stroke expertise among nonstroke unit staff
LOS^f^	Target LOS benchmarks: ischemic stroke: 5 d; hemorrhagic stroke: 7 d No established benchmark for inpatient TIA LOS	Mean stroke LOS: 11.2 d Mean TIA LOS: 4.5 d (financial year 2022-2023)	Excess LOS for stroke admissions (6-day gap relative to best practice), contributing to system inefficiencies
Secondary prevention follow-up	All patients with TIA discharged from the ED should be referred to an SPCg	ED compliance rate of 70%-93%	Inconsistent secondary prevention follow-up; approximately 20% of patients do not receive timely SPC care
Patient and caregiver-centered care	Patients with stroke and their caregivers should be central to care planning and delivery	No formal mechanism to collect stroke-specific patient experience measures	Patient and caregiver perspectives are not systematically informing quality improvement initiatives

^a^ED: emergency department.

^b^ASU: Acute Stroke Unit.

^c^CT: computed tomography.

^d^CTA: computed tomography angiography.

^e^MRI: magnetic resonance imaging.

^f^LOS: length of stay.

^g^SPC: Stroke Prevention Clinic.

As provincial funding policy is beyond our control, we developed the MOTIVE (Mobile Transient Ischemic Attack and Stroke with Adaptive Workflow) team, a targeted QI intervention designed to address local system barriers. MOTIVE deploys a mobile interdisciplinary stroke team to deliver rapid medical and functional assessments at the point of presentation, including in the ED or on nonstroke inpatient units, with a primary focus on patients with TIA or minor stroke. Patients with major or disabling stroke are explicitly excluded and continue to follow the established pathway to the ASU, where stroke unit care remains the standard of care for optimizing outcomes.

The intervention incorporates a dedicated MRI time slot, established through collaboration with diagnostic imaging, to expedite diagnostic decision-making. By decoupling access to interdisciplinary stroke care from mandatory ASU admission for patients who can be safely discharged from the ED [7] while preserving stroke unit care for those requiring inpatient management, MOTIVE is designed to evaluate its impact on LOS, avoidable admissions, support same-day discharge when appropriate, streamline referral pathways, improve patient experience, and align regional practices with the CSBPR.

## Methods

### Study Design

This study is a single-center, prospective, uncontrolled before-and-after QI study, registered prospectively with the Open Science Framework to enhance transparency and minimize duplication [8]. The project follows the Institute for Healthcare Improvement Model for Improvement [9] and is reported in accordance with the SQUIRE (Standards for Quality Improvement Reporting Excellence) 2.0 guidelines [10]. The preimplementation (baseline) period is defined as fiscal year 2022 to 2023 (April 1, 2022, to March 31, 2023), and the postimplementation period covers 12 months following full implementation of the MOTIVE model, with data collected and analyzed at monthly intervals. Implementation will proceed in phased stages over 2 years, with iterative refinement guided by Plan-Do-Study-Act (PDSA) cycles.

Statistical process control methods will be used to evaluate temporal changes in outcomes. Such methods are widely used in health care QI to monitor processes over time and distinguish between common-cause and special-cause variations [11]. Observed changes will be interpreted in relation to PDSA cycles and relevant contextual factors rather than a single fixed intervention point. In this study, statistical process control methods are particularly suitable because the intervention is implemented iteratively and outcomes are expected to evolve over time.

### Eligibility Criteria

The target population for this QI initiative includes all adult patients aged 18 years or older who were presented to the ED or admitted to nonstroke inpatient units with a diagnosis of TIA or acute ischemic stroke and a National Institutes of Health Stroke Scale (NIHSS) score of 5 or less [12]. An NIHSS score of ≤5 was used to define minor stroke. Although patients presenting with an NIHSS score greater than 5, those deemed by the medical team to have disabling deficits, or those who have undergone thrombolysis or thrombectomy are not eligible for MOTIVE assessment at initial presentation, eligibility will be reassessed once bed rest restrictions are lifted and neurological status has stabilized. Patients whose NIHSS score improves to 5 or less at reassessment will become eligible for MOTIVE-led assessment and discharge planning; those who remain above this threshold or continue to have disabling deficits will continue to follow the standard acute stroke care pathway.

Pre-existing cognitive impairment and limited social support (eg, living alone without an identified caregiver) will not be exclusion criteria [13] for the MOTIVE assessment or the evaluation cohort. Rather, these factors inform the interdisciplinary team’s determination of discharge readiness. Patients in whom such factors preclude safe same-day discharge will be managed via the standard inpatient pathway with MOTIVE-led interdisciplinary discharge planning and will remain in the analytic cohort to preserve analytic consistency and selection bias. To assess potential inequities in outcomes among vulnerable patients, a prespecified subgroup analysis will compare primary and balancing outcomes between patients with documented cognitive impairment or those who live alone and the remainder of the cohort.

### Unique Team Roles

The MOTIVE team is led by a stroke neurologist, who holds overall responsibility for medical decision-making and clinical oversight. The team integrates an advanced practice physiotherapist (APP) role into frontline stroke care, a position introduced by HSN in 2021 [14]. HSN was the first academic teaching hospital in Canada to establish an APP role in supporting acute stroke care. The APP role includes functional assessment of patients with suspected or confirmed stroke or TIA, collaborative triage with stroke neurologists and the interdisciplinary team to determine rehabilitation needs and disposition, leadership of QI initiatives, and implementation of evidence-based stroke care practices. This role represents a significant expansion beyond the traditional scope of physiotherapy and necessitates advanced clinical expertise in stroke, training in research and QI, and strong knowledge translation skills. The broader team includes a stroke nurse, occupational therapist, speech-language pathologist, and reactivation worker. The latter is a rehabilitation assistant (reactivation worker) who collaborates with the aforementioned disciplines to facilitate early mobilization and functional recovery of the patient.

### Interest Holders

The MOTIVE team will work collaboratively with the Medical Imaging Department at HSN to optimize access to time-sensitive diagnostics, enabling early discharge. Brain MRI is superior to head computed tomography in diagnostic sensitivity for identifying small ischemic lesions in patients presenting with TIA or minor stroke and can provide additional information to guide decisions regarding diagnosis, prognosis, and treatment. Currently, routine MRI wait times at HSN can extend up to 5 days, which significantly delays the definitive diagnosis and disposition of patients with suspected stroke or TIA. The team will work closely with the medical imaging department to establish “time-cohorted” scheduling whereby urgent MRI scans for MOTIVE patients are prioritized earlier in the day through intelligent reallocation of existing capacity without increasing overall system demand. This approach is anticipated to enable same-day imaging, eliminating the previous multiday wait and allowing stroke neurologists to review the results and confirm final management decisions immediately after the interdisciplinary evaluation is concluded. The team will maintain ongoing discussions with stakeholders, including outpatient therapy services (called outpatient neurological rehabilitation [ONR] at HSN), the Stroke Prevention Clinic (SPC) at HSN, Ontario Health at Home, and other community service providers (such as the poststroke transitional care program run by a community agency) to ensure rapid disposition planning, smooth handover of care, and continuation of evidence-based rehabilitation in the most appropriate setting, whether at home or a community-based program.

### Intervention

The intervention will be fully integrated into routine clinical workflows with the MOTIVE team deployed across the ED and all inpatient units where patients with stroke (except those in the stroke unit) are present. The team will assess 3 specific patient cohorts to ensure early and comprehensive interdisciplinary stroke assessment across all locations within HSN to close existing gaps.

The first cohort includes patients presenting to the ED with suspected TIA or minor stroke. Currently, these patients are typically admitted to a stroke unit for evaluation. The MOTIVE team will conduct a rapid interdisciplinary assessment directly in the ED, facilitating same-day discharge planning for suitable candidates.

The second cohort comprises patients admitted to acute nonstroke inpatient units due to bed shortages, systemic pressures, or stroke resulting from surgeries or other medical conditions during their stay (eg, admitted with cardiac conditions but sustaining a stroke as a complication). Clinical teams in these units often lack stroke-specific expertise, resulting in delays in specialist input and rehabilitation planning for patients. The team will assess all patients with stroke or TIA throughout the hospital (including general medical and surgical floors), ensuring equitable access to best practice stroke care and enabling earlier discharge when clinically appropriate.

The third cohort consists of postacute treatment patients who received intravenous thrombolysis or endovascular thrombectomy and were initially managed in the intensive care unit. Traditionally, these patients are transferred to a stroke unit for interdisciplinary assessment prior to discharge or repatriation. The MOTIVE team will complete a comprehensive assessment in the intensive care unit once patients are medically stable, allowing for direct discharge to home (with or without community support) or repatriation without an intermediate stroke unit stay, thereby reducing the overall LOS at HSN.

These coordinated processes will ensure that all patients with stroke and TIA receive timely expert care, facilitating earlier decision-making, efficient care transitions, and optimized resource use.

### Measurement Strategy

Data will be collected prospectively from administrative databases and electronic medical records by a research assistant and the QI team. All outcomes will be measured and analyzed at monthly intervals to support the longitudinal assessment of system performance over time. As this is a single-center, uncontrolled before-and-after study, baseline and postimplementation cohorts will be compared on key characteristics (age, sex, NIHSS, and stroke type) to assess case-mix comparability. Contextual factors that may influence outcomes (eg, seasonal variation and MRI access) will be tracked monthly, annotated on control charts, and considered when interpreting results. Residual confounding will be acknowledged as a limitation of this study.

The primary outcomes will be (1) the ED-to-inpatient admission rate, defined as the proportion of patients diagnosed with TIA or stroke in the ED who are subsequently admitted to an acute care bed and (2) the acute hospital LOS, calculated as the number of days from admission to discharge on the acute inpatient unit. The secondary outcome will be health care costs, estimated as the number of avoided inpatient bed-days multiplied by the HSN per diem cost.

Process measures will include (1) time from ED presentation to interdisciplinary team assessment, (2) MRI wait time, and (3) the proportion of eligible patients triaged within 24 hours. Balancing measures will include the following:

1. 30-day all-cause readmission rate.
2. The outpatient therapy services (called ONR at HSN) wait time. Data will be gathered monthly by the respective department to evaluate whether the project imposes any additional burden on these wait times. Our QI team does not foresee any issues in this regard, because patients with stroke will access the ONR regardless of their admission.
3. SPC referral volume and wait time will be monitored to ascertain whether admissions avoidance will increase the demand for SPC. As stroke neurologists and stroke nurses initiate secondary prevention measures prior to discharge, SPC follow-up is considered a continuation of care rather than the initial visit.
4. Patient and caregiver experiences will be assessed using a locally developed questionnaire administered at discharge from the hospital, supplemented by an SPC-specific satisfaction survey conducted during patients’ follow-up appointments.

Together, these measures will be used to analyze whether early discharge planning adversely affects patient and caregiver experiences [15].

Patient and caregiver experience will be assessed using a 7-item questionnaire covering five domains mapped to the Donabedian framework [16]: (1) care transitions; (2) diagnostic processes and comfort; (3) patient involvement in decision-making; (4) clarity of information about the condition and medications; and (5) preparedness for discharge, including knowledge of follow-up care. The instrument will be developed by the multidisciplinary QI team through an iterative process, mapping the inpatient stroke journey from admission to discharge, guided by the Donabedian framework and existing literature on stroke-specific patient-reported experience measures [17]. Responses will be measured using a 5-point Likert scale ranging from strongly disagree to strongly agree. Prior to full deployment, the questionnaire will be pilot-tested for clarity and face validity with patients with stroke. It will be administered anonymously in electronic format at the point of discharge; in instances where patients are unable to complete the survey due to cognitive, speech, or physical impairments resulting from stroke, a substitute decision-maker or caregiver will complete it on their behalf. Performance will be evaluated using defect analysis, defined as neutral, disagree, or strongly disagree responses, and top-box analysis, defined as the proportion of respondents selecting strongly agree.

### Ethical Considerations

The HSN Research Ethics Board (file number 25-21) conducted a review of the MOTIVE Project and determined that it is exempt from research ethics board approval. This classification aligns with Article 2.5 of TCPS 2 (2022) [18], as the project is identified as a QI activity. This QI initiative will use data collected for clinical care and routine administrative purposes; therefore, individual patient consent will not be required. Patients will be informed about the use of their data for QI through standard hospital admission processes. All data will be extracted from the HSN electronic medical record and Discharge Abstract Database by authorized institutional personnel. Data will be stored on secure, hospital-managed servers protected by institutional firewalls and accessed only by the designated study team members. Analyses will be conducted on deidentified data, and the results will be reported in aggregate form. The patient experience questionnaire will be administered anonymously, and no identifiable information will be collected or transferred outside the institutional environment.

### QI Tools and Strategies

#### Process Mapping

A stroke neurologist, an APP, program administrative leaders, and frontline clinicians collaboratively developed algorithmic care pathways for patients with suspected TIA or minor stroke in the ED and inpatient settings (Multimedia Appendices 2 and 3) [19].

#### Risk Management

A QI steering committee will be established to manage risks and guide long-term sustainability. The steering committee will comprise individuals with administrative and operational perspectives, including a stroke neurologist, an internal medicine program director, a clinical manager, and a lead physiotherapist. Throughout the project cycle, the team will document both facilitators and barriers encountered during the implementation of this innovative pathway in Northeastern Ontario. Each member will track their individual challenges and the solutions they use. Monthly team meetings and biweekly implementation updates will be conducted to monitor progress, identify barriers, and apply strategies for continuous improvement. This ongoing collaboration will foster continuous learning and adaptation, thereby ensuring the long-term sustainability of the project.

#### Failure Modes and Effects Analysis

A prospective failure modes and effects analysis was conducted to foresee and address potential risks within the MOTIVE workflow prior to its implementation [20]. Informed by the driver diagram (Multimedia Appendix 4) and cause-and-effect analysis, the failure modes and effects analysis focused on system-level vulnerabilities across critical stages of care, including team recruitment and activation, patient identification, interdisciplinary neurological and functional assessment, access to diagnostic imaging, discharge planning, and coordination of community-based services. The anticipated failure modes and their corresponding mitigation strategies are listed in Table 2. During implementation, the relative severity, likelihood, and detectability of these failure modes will be assessed by the MOTIVE team and iteratively refined through regular team meetings using PDSA cycles.

**Table 2:** Anticipated failure modes and mitigation strategies.

Domains from driver diagram	Process step	Anticipated failure mode	Planned mitigation
Recruit and educate team members, define roles, and standardize workflows	Team recruitment and role definition	MOTIVE^a^ team not fully staffed or roles unclear	Early recruitment; written role descriptions (standard of work); standardized workflows
Frontline staff engagement and activation supports	Patient identification (ED^b^ and inpatient units)	Eligible patients with TIA^c^ or mild stroke not identified	Staff education, unit champions, and clear eligibility criteria
Leverage team activation supports	MOTIVE team activation	Delayed or missed team activation	Single-point activation process through the Hypercare software application
Mobile interdisciplinary stroke team	Neurological and functional assessment	Incomplete or delayed neurological and functional assessment prior to disposition	Mandatory stroke neurologist assessment and interdisciplinary (PT^d^, OT^e^, and SLP^f^) assessment
Establish safe discharge criteria	Rehabilitation readiness	Discharge despite inadequate functional independence	AlphaFIM score>80, NIHSS^g^ score <5, and interdisciplinary clinical judgment
Establish coordinated discharge planning strategy	Psychosocial assessment	Social barriers to discharge were not identified	Early screening; social work involvement
Optimize referral pathways for outpatient services	Community service access	Discharge without referral to the Stroke Prevention Clinic, ONR^h^, and Ontario Health at Home	Confirmation that community needs can be met
Patient-centered care and co-design	Disposition, communication, and experience	Discharge decision-making without full interdisciplinary integration, inadequate communication, or negative patient experience, leading to poor engagement and readmission	Interdisciplinary consensus; standardized discharge instructions; patient-reported experience measures; journey mapping; iterative improvement
Timely access to medical imaging and diagnostics	Diagnostic imaging	MRI^i^ was not completed in a timely manner	Designated MRI slots
Organizational culture supports the new model	Sustainability	Decline in adherence to MOTIVE workflows	Quality improvement steering committee oversight

^a^MOTIVE: Mobile Transient Ischemic Attack and Stroke With Adaptive Workflow.

^b^ED: emergency department.

^c^TIA: transient ischemic attack.

^d^PT: physiotherapist.

^e^OT: occupational therapist.

^f^SLP: speech-language pathologist.

^g^NIHSS: National Institutes of Health Stroke Scale.

^h^ONR: outpatient neurological rehabilitation.

^i^MRI: magnetic resonance imaging.

#### Patient Safety Safeguards

Given that the MOTIVE pathway will facilitate same-day discharge directly from the ED, a structured, multilayered safety framework has been integrated into the clinical protocol to mitigate the risk of premature or unsafe discharge. Eligibility for same-day discharge will require the concurrent fulfillment of six predefined discharge readiness criteria: (1) an NIHSS score below 5 in the context of nondisabling neurological deficits; (2) an AlphaFIM score [21] exceeding 80, indicative of functional independence commensurate with safe return to the home environment; (3) a negative Toronto Bedside Swallowing Screening Test [22], confirming the absence of clinically significant dysphagia; (4) formal interdisciplinary consensus, led by the responsible stroke physician and informed by the APP, occupational therapist, speech-language pathologist, and stroke nurse as clinically indicated, confirming that the patient is medically stable, functionally safe for discharge, and that identified rehabilitation goals, caregiver support, and community follow-up services are in place prior to discharge [4]; (5) completion of appropriate neuroimaging alongside an etiological workup to guide the initiation of evidence-based secondary prevention; and (6) documented confirmation of a safe and supported discharge environment, encompassing either the presence of an identified caregiver or the prospective arrangement of community-based follow-up services (SPC and ONR).

Predefined escalation criteria have been established to ensure the timely identification of clinical deterioration and prompt transition to the inpatient stroke pathway. These criteria include an increase in the NIHSS score from baseline, the emergence of any new disabling neurological deficit, medical or hemodynamic instability, or failure of the bedside swallowing screen. The presence of any single escalation criterion will be sufficient to preclude same-day discharge and initiate inpatient management under the standard stroke unit pathway.

All patients discharged via the MOTIVE pathway will receive a scheduled appointment at the SPC. The safety profile of the MOTIVE pathway will be prospectively monitored through a set of predefined balancing measures, including the 30-day readmission rate and the rate of ED revisits within 7 days of discharge. The comprehensive discharge readiness criteria and corresponding escalation framework are presented in Textbox 1.

**Discharge criteria.**

**Inclusion criteria**

- Transient ischemic attack or mild stroke (National Institutes of Health Stroke Scale [NIHSS] score <5) and nondisabling stroke
- Assessment completed by stroke neurologist
- Functional assessments completed by a physiotherapist, occupational therapist, and speech-language pathologist to determine the types of therapy required based on identified deficits
- Functional deficits assessment using the AlphaFIM score (>80), with the patient's ability to attend outpatient neurorehabilitation independently or with caregiver support
- The patient is medically stable
- The patient's present medical, personal care, and rehabilitation needs can be effectively managed in the community
- No dysphagia on clinical screening and standardized swallowing assessment (The Toronto Bedside Swallowing Screening Test)
- Patients admitted primarily because of social circumstances (eg, housing concerns, caregiver support breakdown, or difficulty managing at home) will be assessed on an individual basis. The stroke team will collaborate with a social worker when social barriers to discharge are identified. If these barriers can be addressed promptly, discharge directly from the emergency department (ED) may be considered.

**Important considerations**

- NIHSS and AlphaFIM scores are only one component of discharge planning. Final discharge decisions require a collaborative assessment by the stroke neurologist, stroke nurse, physiotherapist, occupational therapist, and speech-language pathologist. For example, a patient with a posterior circulation stroke may have a low NIHSS score but still exhibit disabling truncal or gait ataxia requiring admission. Conversely, a patient with an AlphaFIM score >80 may have significant cognitive deficits that warrant inpatient assessment and treatment.
- If a speech-language pathologist is unavailable in the ED, the assessment will be completed in the inpatient setting for selected patients with clinically significant language impairment or dysphagia.

**Exclusion criteria**

- Moderate to severe stroke (NIHSS score >5)
- Medically unstable
- AlphaFIM scores < 80
- Unable to access community rehabilitation services (eg, outpatient neurological rehabilitation or Ontario Health at Home)
- Complex social problems

#### Discharge Criteria

All patients with TIA or stroke will have a follow-up appointment at an SPC for prevention management. Patients with stroke with minor impairments in any domain (physical, cognitive, speech, or psychosocial) will be referred to appropriate community rehabilitation services (ONR, Ontario Health at Home, and vision loss rehabilitation services) prior to discharge. We adopted the CSBPR recommendations for a safe discharge plan to ensure that patients with stroke receive the best rehabilitation care and expedite their recovery [5]. The criteria for safe discharge from the ED or inpatient units to home are based on several factors (Textbox 1).

### Sample Size Calculation

One of the aims of the MOTIVE QI project is to implement an earlier, coordinated discharge plan [23], thereby reducing the overall LOS. We calculated the required sample size for this single-arm QI project based on the baseline data extracted from the Discharge Abstract Database. Our 2022 baseline data, comprising 278 patients with ischemic stroke, revealed an average LOS of 11.2 days with an SD (σ) of 18.27 days. The steering committee’s initial goal was to achieve a 30% reduction from this baseline, targeting an average LOS of 7.84 days. It represents a minimum detectable change (Cohen *d*) of 3.36 days. We will use continuous real-time monitoring to identify barriers and will use PDSA cycles to align our target with the provincial benchmark of 5 days [6]. To detect this initial improvement, a sample size calculation [24] indicated that 183 patients were required, with a statistical power (β) of 80% and a significance level (α) of 5%.

## Results

This is a study protocol; therefore, results are not available. The planned analyses will use statistical process control methods to track key indicators over time using run and control charts appropriate for the data type. Such methods are widely used in health care QI to monitor processes and distinguish between common-cause and special-cause variation [11,25]. Indicators will be plotted monthly using run charts during the early phases and appropriate control charts thereafter, including individual and moving range charts for continuous variables and p-charts for proportions (eg, admission and readmission rates).

The data will be reviewed monthly with annotated timelines for the PDSA cycles to assess their impact. Admission rates from the ED to acute care beds, 30-day readmission rates with the same diagnosis, referral rates to SPC, and outpatient therapy services will be analyzed using the chi-square test. Differences in LOS before and after implementation will be examined using parametric or nonparametric tests as appropriate, based on data distribution. Home time will be calculated using *t* tests or Mann-Whitney *U* tests based on the distribution of characteristics.

## Discussion

This protocol outlines the implementation and evaluation of an interdisciplinary mobile stroke team assessment model designed to address delays in early rehabilitation screening and discharge planning at a regional stroke center in Northeastern Ontario. The MOTIVE intervention responds to well-recognized system challenges in stroke care, including ED overcrowding, prolonged LOS, and delayed discharge planning arising from limited access to early interdisciplinary assessments outside dedicated stroke units. By delivering rapid assessments at the point of care and facilitating timely access to diagnostic imaging, the model is designed to improve system efficiency while maintaining alignment with evidence-based stroke care.

The MOTIVE team comprises a stroke neurologist, APP, occupational therapist, speech-language pathologist, reactivation worker, and stroke nurse, and provides comprehensive assessments across multiple care settings, including the ED and nonstroke inpatient units such as intensive care, medical, surgical, cardiac, and oncology wards. The intervention targets 3 key patient groups: individuals presenting to the ED with TIA or minor stroke, inpatients who develop stroke symptoms while admitted to nonstroke units, and patients treated after thrombolysis or thrombectomy. By conducting timely medical and functional assessments at the point of presentation, the model is intended to support early discharge, reduce potentially avoidable admissions, and facilitate direct transitions from acute care settings to home with appropriate outpatient or community follow-up.

A novel component of this intervention is the expanded frontline role of APPs [14]. This role supports the early triage of rehabilitation needs in collaboration with an interdisciplinary team, contributes to QI leadership, and facilitates continuity between acute and community-based services. In addition, structured collaboration with diagnostic imaging, including time-cohorted MRI access, and engagement with community partners are designed to support timely clinical decision-making without increasing the overall system demand.

Guided by the Institute for Healthcare Improvement framework and iterative PDSA cycles, the project will examine changes in ED admission rates, LOS, 30-day readmissions, and patient satisfaction. Cost implications will be explored by estimating the avoided inpatient days. Potential implementation challenges include workforce availability, patient complexity, and reliance on community service capacity, which will be monitored as part of the QI process.

If feasible and sustainable, the MOTIVE model may inform the development of mobile interdisciplinary stroke team assessment approaches in other hospital settings. The findings of this evaluation will contribute to the growing evidence base on system-level interventions aimed at improving access, efficiency, and patient-centered care across the stroke continuum.

To conclude, the MOTIVE model introduces an innovative interdisciplinary mobile assessment approach for patients with TIA and stroke in Northeastern Ontario. By providing rapid evaluations in the ED and nonstroke inpatient units, it directly addresses ED congestion, prolonged LOS, and delayed discharge planning. If successful, this scalable, patient-centered framework has the potential to set a new standard for efficient, cost-effective stroke care in urban and rural Canadian hospitals.

## Appendix

Multimedia Appendix 1 Cause-and-effect diagram.

Multimedia Appendix 2 Process map emergency department pathway.

Multimedia Appendix 3 Process map inpatient pathway.

Multimedia Appendix 4 Driver diagram.

## Abbreviations

APP: advanced practice physiotherapist
ASU: Acute Stroke Unit
CSBPR: Canadian Stroke Best Practice Recommendations
ED: emergency department
HSN: Health Sciences North
LOS: length of stay
MOTIVE: Mobile Transient Ischemic Attack and Stroke with Adaptive Workflow
MRI: magnetic resonance imaging
NIHSS: National Institutes of Health Stroke Scale
ONR: outpatient neurological rehabilitation
PDSA: Plan-Do-Study-Act
QI: quality improvement
SPC: Stroke Prevention Clinic
SQUIRE: Standards for Quality Improvement Reporting Excellence
TIA: transient ischemic attack
