# Supplementary figures and images for: An Innovative Model of Stroke Care for Rapid Assessment and Discharge of Patients With Transient Ischemic Attack and Stroke in Northeastern Ontario: Protocol for the Implementation and Evaluation of MOTIVE (Mobile Transient Ischemic Attack and Stroke With Adaptive Workflow) Team
Source: JMIR Res Protoc. 2026 Jul 3;15:e93315. doi: 10.2196/93315 (PMC13379694; doi:10.2196/93315)

# Cause-and-effect diagram

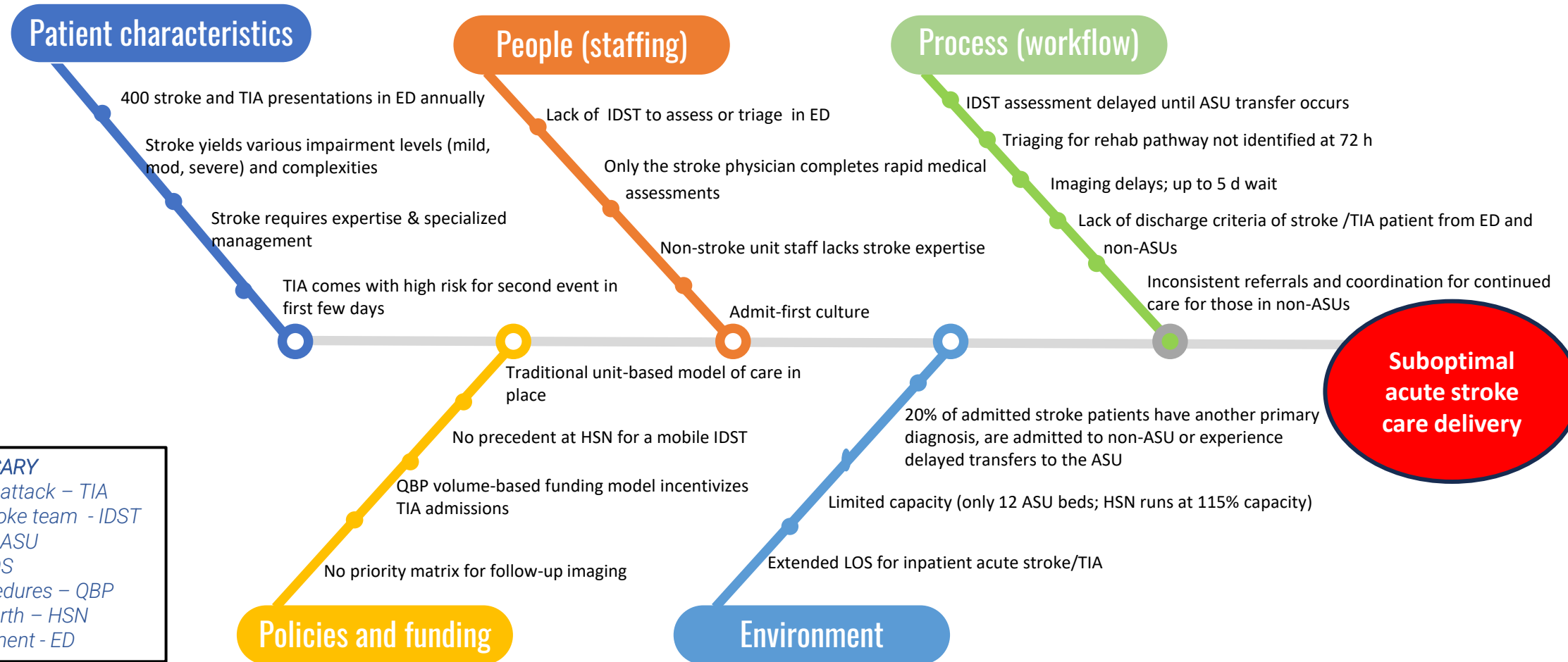

Supplement: Multimedia Appendix 1 [file resprot_v15i1e93315_app1.pdf]

# Process Map

## ED pathway

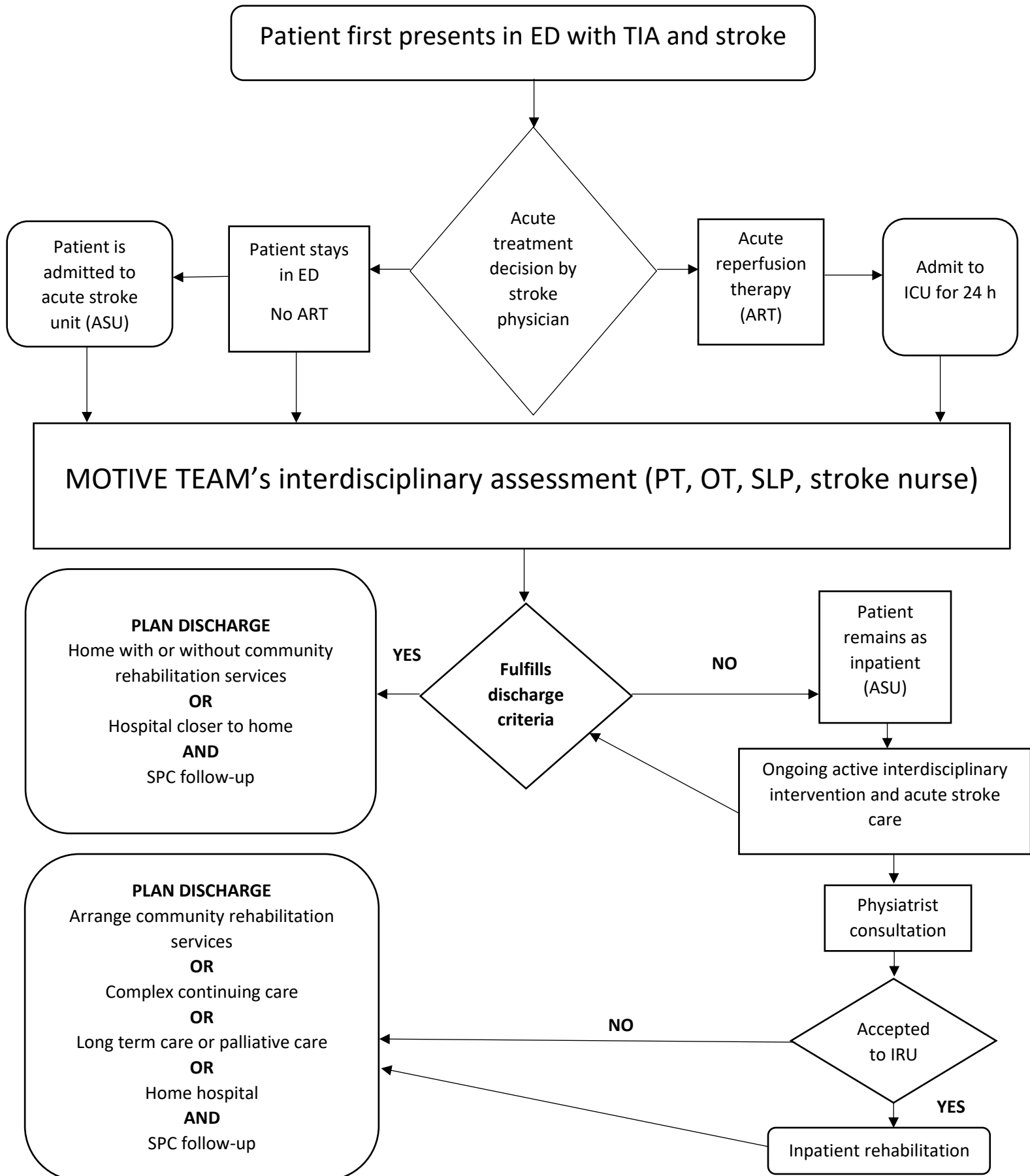

Supplement: Multimedia Appendix 2 [file resprot_v15i1e93315_app2.pdf]

# Process Map

## Inpatient pathway

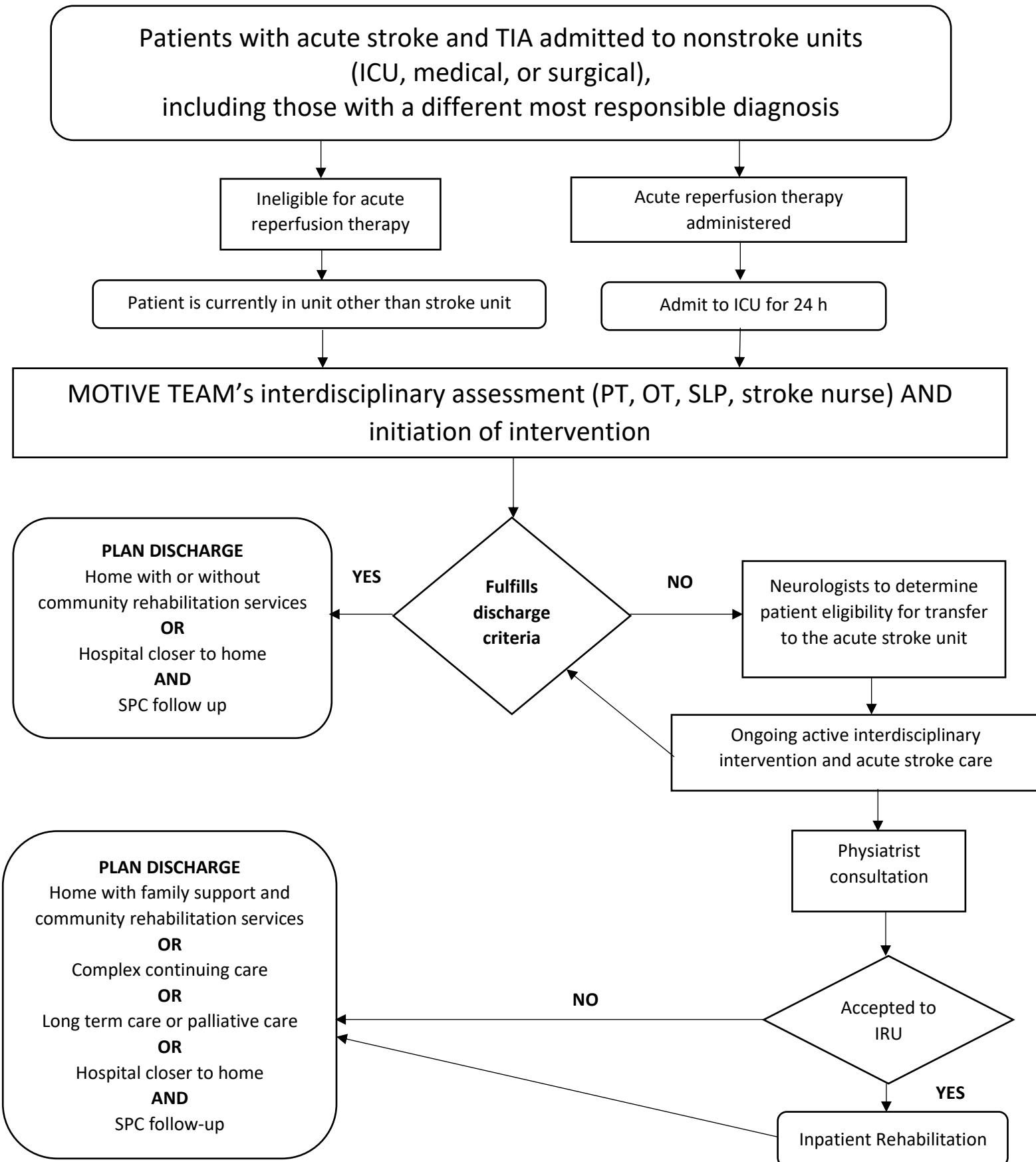

Supplement: Multimedia Appendix 3 [file resprot_v15i1e93315_app3.pdf]
