# Supplementary material for: An Innovative Model of Stroke Care for Rapid Assessment and Discharge of Patients With Transient Ischemic Attack and Stroke in Northeastern Ontario: Protocol for the Implementation and Evaluation of MOTIVE (Mobile Transient Ischemic Attack and Stroke With Adaptive Workflow) Team
Source: JMIR Res Protoc. 2026 Jul 3;15:e93315. doi: 10.2196/93315 (PMC13379694; doi:10.2196/93315)

Driver diagram

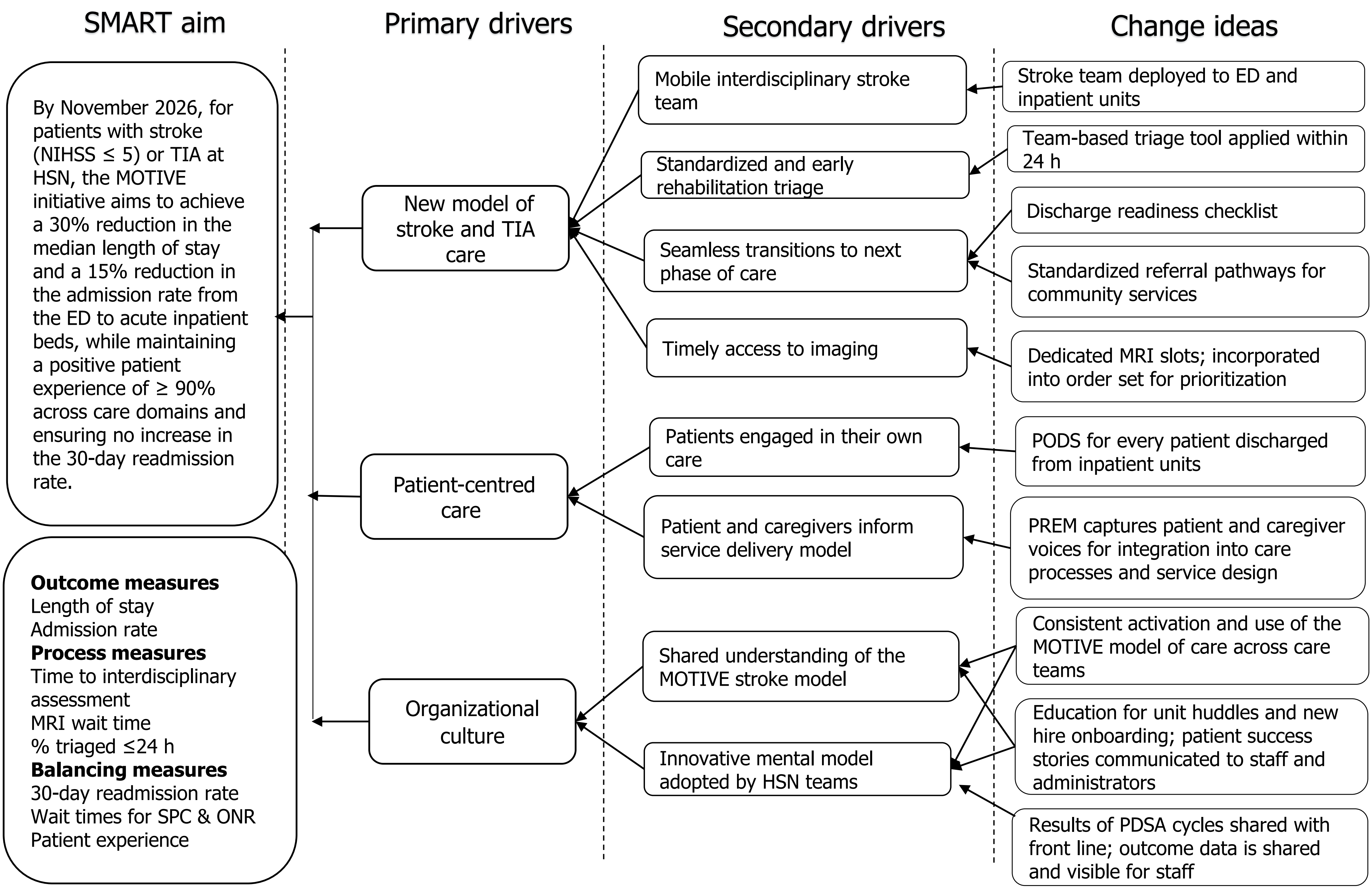

Supplement: Multimedia Appendix 4 [file resprot_v15i1e93315_app4.pdf]
